# Two-step diffusion in cellular hygroscopic (vascular plant-like) materials

**DOI:** 10.1126/sciadv.abm7830

**Published:** 2022-05-13

**Authors:** Marion Cocusse, Matteo Rosales, Benjamin Maillet, Rahima Sidi-Boulenouar, Elisa Julien, Sabine Caré, Philippe Coussot

**Affiliations:** 1 Laboratoire Navier (Ecole des Ponts Paris Tech-Univ Gustave Eiffel-CNRS), Champs-sur-Marne, France.; 2 Experimental Soft Condensed Matter Group, School of Engineering and Applied Sciences, Harvard University, Cambridge, MA, USA.

## Abstract

Vascular plants, a vast group including conifers, flowering plants, etc., are made of a cellular hygroscopic structure containing water in the form of either free (i.e., in a standard liquid state) or bound (i.e., absorbed in the cell walls) water. From nuclear magnetic resonance techniques, we distinguish the dynamics of bound water and free water in a typical material (softwood) with such a structure, under convective drying. We show that water extraction relies on two mechanisms of diffusion in two contiguous regions of the sample, in which respectively the material still contains free water or only contains bound water. However, in any case, the transport is ensured by bound water. This makes it possible to prolong free water storage despite dry external conditions and shows that it is possible to extract free water in depth (or from large heights) without continuity of the free water network.

## Teaser

Capillary and absorbed (bound) water extraction from vascular plants relies on two-step diffusion process governed by bound water.

## INTRODUCTION

Water transport (drying, absorption, and sap flow) is fundamental in the life or use of natural materials such as plants, trees, wood, vegetables, fruits, seeds, etc. Most of these systems are made of a cellular hygroscopic structure containing water molecules with different mobilities as observed by nuclear magnetic resonance (NMR) relaxometry (*1*–*12*). Typically, free water is contained in cavities several orders of magnitude larger than the molecular scale, while bound water is set through hydrogen bonds between lignocellulosic macromolecules. In wood, some of these cavities may be connected via pits or nanopores, possibly allowing capillary transport such as assumed in the tension-cohesion theory for sap flow (*13*, *14*), but these pits are tiny and few, and for the other material types abovementioned, there are no such connections so that water transport must rely on exchanges between bound and free water. These characteristics make the liquid transport processes in all these materials rather complex (*15*).

For example, for wood drying, it has often been considered that, in a first stage, some free water is withdrawn from the sample by capillary forces, while in a second stage, drying essentially occurs by diffusion of vapor and/or bound water through the sample (*16*–*18*). However, the detailed characteristics of these two phases and the conditions of transition between them are not well identified or experimentally proven by internal observations. In this context, basic models essentially provided a phenomenological description of drying when the wood sample only contains bound water, assuming different possible driving forces [vapor pressure, moisture content (MC), chemical potential, etc.] at the origin of the process (*19*, *20*). More sophisticated modeling approaches taking into account different transport mechanisms for bound water, free water, and water vapor have later been developed (*21*–*25*). They appear to be able to predict macroscopic trends as soon as a number of parameters are introduced and possibly fitted. However, in these approaches, the transport of free and bound water is still considered separately, i.e., the exchanges (fluxes) or their coupling (interaction) are not particularly taken into account. Such possible coupling does not seem either to be taken into account in drying models for fruits or vegetables, but in that case, shrinkage can also play a major role (*26*).

Yet, in recent years, internal measurements suggested some coupling between the water in these two states, partly or fully governing the drying dynamics. In particular, it was shown that bound water controls the imbibition in most wood types, i.e., in hardwood (*27*, *28*) (wood from angiosperms, including a group of flowering plants) and softwood (*2*) (wood from gymnosperms, including conifers). Free water appears to progress only when the cell walls contain a sufficient amount of bound water, which strongly damps the expected capillary imbibition (typically by a time factor of 1000). For drying, different phenomena were observed: in some cases, a homogeneous decrease of the water content throughout the medium and, in other cases, a dry front developing from the free surface (*29*–*32*). In addition, it was observed that during slow drying, the bound water concentration decreases when most free water has disappeared (*31*–*33*), while for fast drying in hardwood, the bound water content decreases proportionally to the free water content (*4*). To explain the latter effect, it was suggested that the free water is extracted by absorption in the cell walls and then transported toward the free surface (*1*). However, as yet, there is no direct observation of the bound water transport through the structure and thus no quantification of the process allowing to develop and validate modeling approaches.

**Fig. 1.**
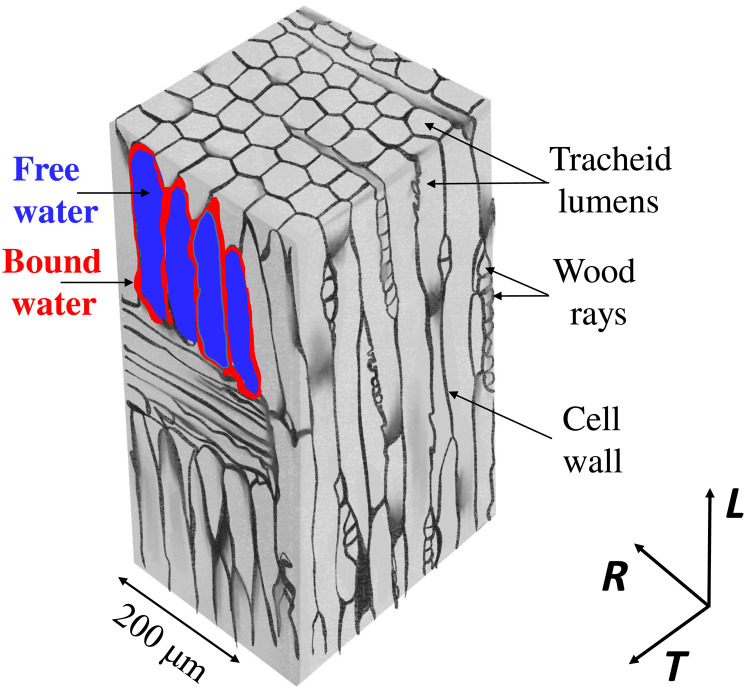
Typical basic structure of Douglas fir wood. Scheme drawn from microtomograph image extract (*70*). The possible location of bound and free water is illustrated in a limited region. Note that only the macroporosity may be seen in such a scheme. The axes indicate the wood anisotropic directions: *L*, longitudinal (tree axis); *R*, radial; *T*, tangential.

Here, we propose a detailed view of the internal mechanisms of drying in a typical cellular hygroscopic material, i.e., a softwood (Douglas fir). Its structure is basically made of tracheids (from 20 to 50 μm for Douglas), which are tubular cells along the longitudinal direction (i.e., tree axis) of millimetric length (see Fig. 1). Tracheids may be connected to each other through pits that contain very small openings, whose size ranges from a few nanometers to a few tens of nanometers depending on species and samples (*34*). These pits may be open or closed depending on wood sample hydric history and localization in the trunk or in the ring. The structure also contains rays, which are long conduits (in the radial direction of the tree) of diameter ranging from 5 to 10 μm. To sum up, the structure is a cellular network of cavities with tiny connections between them. Tracheids and rays can contain bulk water in a standard liquid state, i.e., free water (see Fig. 1). The solid porous network, composed of cell walls, can also contain water (see Fig. 1) placed between the hydroxyl groups of lignocellulosic wood components to form hydrogen bonds. This bound water is spontaneously absorbed by the structure placed in contact with liquid, as long as the maximum MC associated with bound water alone [i.e., the so-called fiber saturation point (FSP)] is not reached.

Such a structure exhibits the basic characteristics of vascular plants (*35*), which is a vast group of plants including gymnosperms, angiosperms, and various other species: a hygroscopic solid cellular porous network, which tends to absorb water in the cell walls and contains free water in cavities (lumens). Thus, although the detailed geometry of the structure varies from one system to another, their basic characteristics in terms of water transport (i.e., free water in cavities, bound water in cell walls, and possible exchange between both water types) are similar, so that we can consider our wood sample as a model material of a wide range of plant-like systems.

With the help of NMR, we first show that, as for hardwood (*1*), in softwood, free water is extracted only by absorption as bound water in the cell walls; then, it is transported in this state through the material. By using magnetic resonance imaging (MRI), which offers a more detailed quantification of spatial dynamics of bound and free water separately, along with modeling, we then show that water extraction relies on two mechanisms of diffusion: a first process with a small diffusion coefficient when the material still contains free water; a second one, with a much larger diffusion coefficient, when there is only bound water. Depending on the boundary conditions (air flux), the material responds in different ways. For a sufficiently weak air flux along the free surface, the free water is extracted homogeneously from the sample and a constant drying rate is observed; for a sufficiently strong air flux, a region without free water soon develops, and the interface between this region and the region containing free water propagates in the material with the square root of time, but water diffuses through both regions. As a result of this process, in this case, the drying rate is limited by the diffusion process so that further increasing the air flux intensity does not change it. These results allow to rationalize and explain various previous observations (*1*, *4*, *32*) on the macroscopic evolutions of bound and free water during drying.

Last, we demonstrate that even when subjected to a strong dry air flux, such natural systems control and limit the rate of extraction of water. This effect could constitute a self-protective system, which makes it possible to prolong free water storage despite dry external conditions. We thus show that it is possible to extract free water in depth in the material without continuity of the free water network. This full description and characterization of the internal processes open the way to a simple physical description of drying and provide key elements for a general understanding of water transport in various hygroscopic porous systems.

## RESULTS

### General internal characteristics of drying

**Fig. 2.**
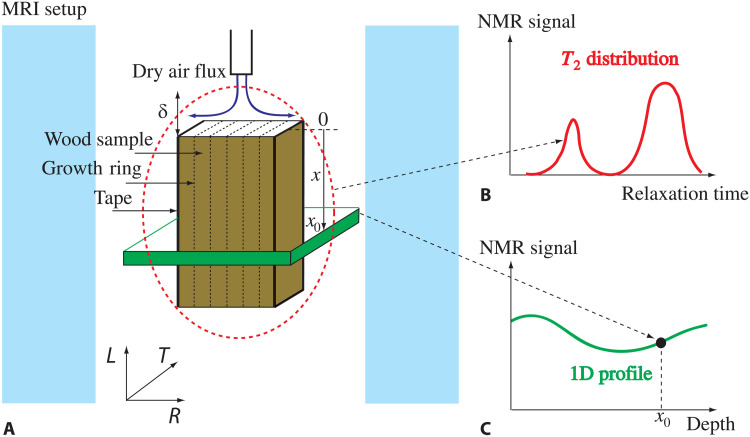
Experimental setup and measurement techniques used. Scheme of the experimental setup with the sample inside the magnet (**A**) and main NMR (*T*_2_ distribution) (**B**) and MRI (1D profile) (**C**) sequences used to explore the water content during sample drying.

We first look at the general characteristics of the water evolution in wood sample subjected to different air flux intensities, inside MRI systems (see Fig. 2A) (see Materials and Methods). The samples were covered with an impermeable tape on all sides except the bottom surface (lying on the container bottom) and the top surface. Under these conditions, water can be extracted in the form of vapor only through the upper free surface, which corresponds to an RT plane (see Fig. 2A). We thus here look at the drying in the longitudinal direction, i.e., along the main axis of the tracheids. The air flux imposes a vapor diffusion from the wet regions of the sample to the dry air zone, which constitutes the boundary condition along the free surface of the sample. However, it is not so easy to translate this boundary condition in terms of drying rate or water distribution, as these characteristics evolve as a result of the interaction between the wetness of the sample and the air flux. Moreover, the impact of the air flux depends on the exact flux orientation and position and on the shape and roughness of the sample surface. Under these conditions, in the first part of the paper, we will simply consider “weak” and “strong” air fluxes in a qualitative way, essentially through their impact on drying characteristics, and without detailing the exact air flow conditions. We will be more precise on this boundary condition in the last part of the paper, where it will be shown possible to describe and quantify it in a straightforward way through a single parameter expressing the characteristic thickness of the zone of vapor exchange along the sample surface (see below).

We measured the distribution of relaxation time *T*_2_, which is the probability density function of NMR relaxation times in the whole sample (see Fig. 2B). It is worth noting that in such measurement, the signal of any water molecule is taken into account (see Materials and Methods). Roughly speaking, this relaxation time is related to the mobility of water molecules and specific interactions of water with their environment (e.g., adsorption, proton exchange with other species, or magnetic interactions at nanoscale). In the particular case of water embedded in a pore, within the usual hypothesis of biphasic fast exchange (*36*), the relaxation time scales as the ratio of the volume of free liquid water to the area of the water-solid interface, with a factor depending on the NMR surface relaxivity (which depends on the physicochemical properties of the interface).

**Fig. 3.**
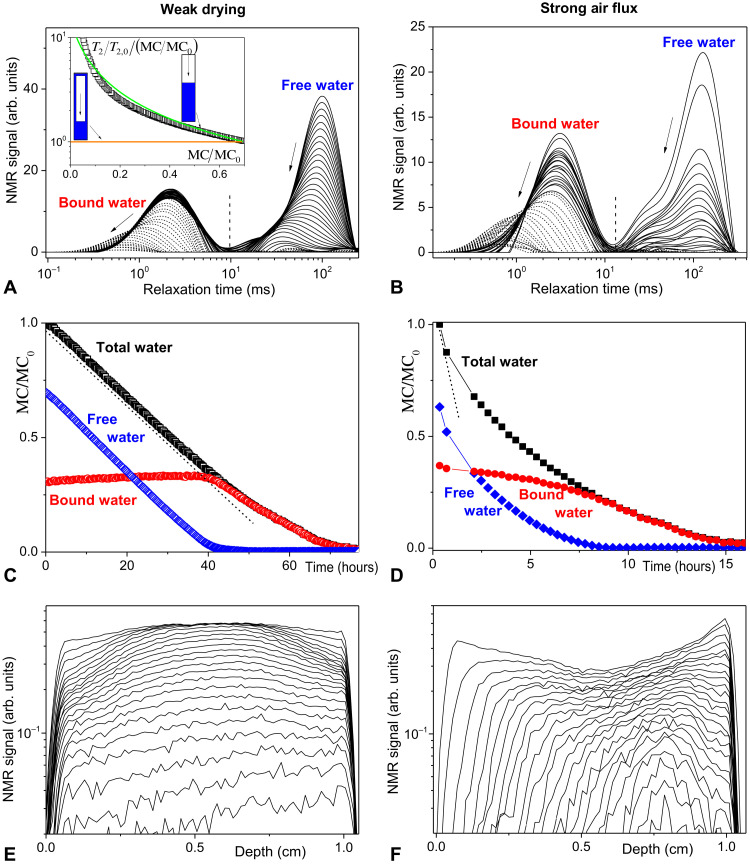
Internal MC characteristics during wood drying. Water transfers during drying of wood sample under weak (**A**, **C**, and **E**) or strong (**B**, **D**, and **F**) dry air flux, with 1-cm-long samples of respective initial MC 67 and 62%: (A and B) relaxation time distributions [(A) every 80 min; (B) at 21, 42 min, and then, every 21 min from 2 hours and 7 min), the arrows indicate the time increase, the distributions represented by continuous lines correspond to the period during which free water exists in the sample, the distributions represented by dashed lines to the next period; (C and D) evolution of each water type as a function of time, as deduced from the integration of the distributions over the corresponding range of relaxation times [see separation dashed lines in (A and B)] and expressed as the fraction of water mass relatively to the initial total water mass; the resulting mass flux in the constant drying rate period is 2 × 10^−5^ kg m^−2^ s^−1^; note that the time scales are not the same for the two tests; the straight dashed lines are guides for the eyes; (E and F) 1D distribution profiles of free water in time, successive profiles from top to left correspond to successive times [every 105 min for (E) and every 21 min for (F)]. The dry air flux is imposed along the free surface of the sample situated at a zero depth. The inset in (A) shows the measured ratio (squares) of average relaxation time over the free water domain to MC (rescaled by initial value) as a function of the MC. This ratio computed for two model situations (cylindrical tracheid, of length five times its diameter) is also shown by continuous lines: (top) dewetting and (bottom) no dewetting.

From the peaks appearing in the *T*_2_ distributions (see Fig. 3, A and B), we can distinguish different water characteristics, associated with its distribution inside the specific structure of the sample. During drying, the amplitude, position, and shape of these peaks can change, reflecting the evolutions of the water content, the geometrical distribution of the water in pores, and the changes in pore size or shape. The main peak on the right, i.e., for a *T*_2_ around 100 ms, corresponds to water in the largest pores, i.e., in tracheid lumens. The peak on the left, say around 2 to 3 ms, corresponds to less mobile water, i.e., bound water. There is also a small peak situated around 20 ms, which likely corresponds to water in rays whose size is a few times smaller than the tracheid diameter. However, this (ray) peak cannot always be identified, as it is close to the larger peak of free water in tracheid lumens, which tends to slightly damp it and shift it toward higher *T*_2_ values [an effect resulting from the Laplace transform procedure used for data analysis (see Materials and Methods)]. We have no means to strictly distinguish the water amount in rays and tracheids from the analysis of such a distribution. However, we can remark that they seem to evolve approximately in a similar way in time. For these reasons, in the following discussion, we will simply consider these two peaks as a whole and refer to them as the free water fraction (whose main fraction is in tracheid lumens).

During the same experiments with our NMR spectrometer, we also measured (see Materials and Methods) the spatial distribution of free water along the sample axis, which gives successive profiles of NMR signal as a function of depth in time. We thus get one-dimensional (1D) profiles in which each point represents the total NMR signal associated to free water in a thin cross-sectional layer of the sample (see Fig. 2). In our case, the thin layer has a thickness of either 0.125 or 0.75 mm depending on the measurement system used.

### Weak air flux

#### Observations

For a sufficiently weak air flux tending to impose a slow drying (see Fig. 3, A, C, and E), during a first stage, we observe that the amplitude of the peaks associated with free water in tracheids and rays decreases over time until complete disappearance. Its position along the *T*_2_ axis initially slightly varies in a first period, and then

it moves toward substantially lower values. In the next stage, which starts as soon as the peak of free water has disappeared, the peak associated with bound water starts to move toward lower *T*_2_ and decreases in amplitude.

The amounts of free and bound water in time may be estimated from the integration of the *T*_2_ distribution in the range of relaxation times corresponding to each water type (see Fig. 3, C and D). As long as the relaxation time of most water phases in the sample lies in the range covered by our measurements, the integral over all times is proportional to the total water mass. For Douglas wood, the validity of this statement was for example checked by successive comparisons during imbibition (*2*). The total mass of water is described via the MC (symbol MC), which is the ratio of the mass of water to the dry mass (*m*) of the sample. The other water type amounts are described through the fraction of MC they represent. For the weak air flux, the variations of the different water types confirm that the bound water starts to disappear only when all the free water has been removed (Fig. 3C). The water mass versus time during the first period is a straight line, which means that the drying rate is constant as long as the sample is saturated with bound water and still contains some free water. Note that there is an apparent slight increase of bound water amount during the first period (i.e., when free water is present), which can hardly correspond to a real physical phenomenon. This is an artifact due to the treatment procedure involving a Laplace transform: As the free water peak decreases, the amplitude of the neighboring (bound water) peak can slightly increase. Thus, actually, the bound water amount should be considered as constant during the first period.

At last, we can see that the 1D profiles of the free water distribution (see Fig. 3E) remain essentially horizontal. This means that the free water content decreases homogeneously throughout the sample. Since locally, because of the fast absorption of free water in cell walls, wood is saturated with bound water as soon as it contains free water, we deduce that the distribution of all water is also homogeneous.

#### Analysis

The critical features of the slow drying as observed by NMR are as follows: As long as there is free water in the sample, (i) the drying rate is constant, (ii) the water distribution is homogeneous, and (iii) the relaxation time of free water slowly decreases. From these features, we can deduce some fundamental mechanisms of wood drying.

A constant drying rate period tells us much about the drying mechanism. Since drying results from water evaporation whose rate depends on the gradient of relative humidity (RH) (through the Fick’s law) around the region of evaporation, a constant drying rate means that the humidity conditions remain approximately constant in this region, and the interaction with the air flux does not vary (see below). Note that since the drying rate is observed from the beginning of the test, this region of evaporation is around the sample free surface. As a corollary, this means that the water is continuously transported toward the free surface, from the inner sample region.

A homogeneous distribution of water in the sample all along the process is the hallmark of some powerful physical effect, which maintains at equilibrium the water concentration throughout the sample. Actually, this effect plays a critical role in the constancy of the drying rate: As long as there is free water, the sample is saturated with bound water so that the RH in the air in contact with any point of the wood surface is close to 100%; this, in particular, means that the boundary conditions for vapor transport do not change, and the evaporation rate is constant. Moreover, keeping a homogeneous distribution while the sample bottom is dead-end implies a continuous transport of water toward the sample free surface.

In various porous media (*37*) such as bricks or sandstone (*38*), pastes (*39*), or granular packings (*40*–*42*), a constant drying rate period is also observed and associated with a homogeneous desaturation of the simple porous media. In those cases, the Laplace pressure (pressure drop through the air-liquid interface) ensures a continuous capillary reequilibration of the liquid network throughout the medium, which levels the saturation (water to pore volume ratio) and induces a net liquid transport toward the free surface where it can evaporate. Then, the constant drying rate results from the fact that the RH remains around 100% as long as a sufficiently large density of liquid patches exists close to the free surface (*43*). It is worth emphasizing that such a behavior is essentially observed for a liquid well wetting the solid (i.e., contact angle substantially smaller than 90°), so that the minimization of the air-liquid interface can govern the liquid distribution. Otherwise, i.e., for a partially wetting liquid, the extraction of some liquid by evaporation from the free surface will preferentially lead to the dewetting of the upper layers. Then, a dry region will develop immediately, associated with a decrease of the drying rate (*44*) since the evaporation now occurs at an increasing distance from the sample free surface and the vapor has to diffuse over a longer distance before reaching the dry air region. Local variation of wettability can lead to more complex effects (*37*).

For media such as granular packings, another important factor at the origin of this continuous capillary reequilibration throughout the medium is a well-connected porous network with a continuous distribution of pore sizes. This allows for a progressive homogeneous desaturation of the medium associated with a continuous homogeneous increase of the Laplace pressure, leading to the successive reequilibration effects.

This contrasts with a (model) material in the form of a network of large pores connected by small openings. In that case, air can enter a large pore only if a liquid-air interface with a curvature radius smaller than the opening size transiently forms. The resulting Laplace pressure being, in general, much larger than that associated with a partially empty pore leads to an almost instantaneous invasion of the pore by air. As a consequence, the air entrance essentially occurs in the form of successive invasions of neighboring pores, a process reminiscent of the concept of invasion percolation but more precisely described with the help of pore network models (*45*, *46*), in which large pores are connected by throats of different (small) sizes. This situation is similar to that assumed to prevail during the very first stage of embolism in wood resulting from droughts (*47*, *48*), but the possible next stage, i.e., invasion of air at larger distance, has apparently not been much studied.

Such a scheme, i.e., successive invasion by air of pores (here mainly tracheids), is logical considering the softwood structure made of relatively large pores, i.e., tracheids and rays, connected by tiny openings, nanopores, or weak seals. However, it brings up two major issues: According to this scheme, in strong contrast with our observations, the saturation would rapidly become strongly heterogeneous in the sample, and the drying rate would not remain constant as the evaporation surface would recede in the sample.

We are thus led to further consider the possibility of a continuous liquid network persisting throughout the medium, to ensure some capillary reequilibration process, as in a simple porous medium (see above). Considering the specific structure of wood, this would mean that liquid films would remain along the cell walls of tracheids invaded (partially) by air, connecting filled or partially emptied tracheids.

Actually, we have some straightforward evidence of the fact that, more generally, a continuous liquid network is not maintained. This comes from the slow variation of the relaxation time observed for the free water (see Fig. 3A). This observation provides a fundamental information on the interaction between the free water and the cell wall surfaces and, consequently, on the shape of these liquid volumes. The relaxation time of water molecules in a large volume is in the order of 2 s, but it is in general smaller by several orders of magnitude for molecules along a solid surface, here, the cell walls. As a consequence, the final relaxation time of molecules depends on the respective times they spend along the surface and in the bulk. Here, in a tracheid, the mean square distance covered by a water molecule in a cross section as a result of self-diffusion during *T*_2_ = 100 ms (initial average value over the corresponding peak curve) is $\sqrt{4{\mathrm{DT}}_{2}}\approx 30\;\mu m$, with *D* ≈ 2 × 10^−9^ m^2^ s^−1^ the self-diffusion coefficient of water, which is about twice the tracheid radius. It follows that, over this time, most molecules have at least a few opportunities to reach the wall, so that the resulting relaxation time is substantially influenced by the time spent on average by molecules along the solid surface. It is possible to be more precise under the assumption of the fast-diffusion model of Brownstein and Tarr (*36*), which predicts that the relaxation time *T*_2_ for free water is proportional to the ratio of the liquid volume (proportional to MC) to the area of the liquid/solid interface in the pores. Thus, the evolution of the ratio of the relaxation time to the MC, i.e., *T*_2_/MC, which according to this model is inversely proportional to the area of the liquid/solid interface, should tell us much about the wetting conditions during drying. If the liquid dewets the cell walls, the surface of contact between free water and lumen decreases so that *T*_2_/MC will increase substantially. On the contrary, in the absence of dewetting, *T*_2_/MC will remain constant. In our case, *T*_2_/MC substantially decreases, which implies a dewetting. More precisely, we can remark that *T*_2_/MC evolves rather closely (see inset of Fig. 3A) to the theoretical prediction for a direct dewetting of a cylindrical tracheid according to the Tarr and Brownstein model.

Since the water distribution remains homogeneous throughout the sample while the MC decreases, this dewetting necessarily induces a disconnection of the liquid network, which evolves in the form of isolated liquid volumes in the lumens. Last, we expect a water distribution in the form of liquid volumes along the cell walls or at the end of the tracheids and with a characteristic size similar to the tracheid diameter. This situation exactly corresponds to that observed from x-ray computed tomography in drying poplar (*1*).

As a consequence, the liquid network is disconnected, whereas the water distribution remains homogeneous up to the very last amount of free water (see Fig. 3E). This clearly differs from drying in granular packings, in which the drying rate starts to decrease for a saturation of 10 to 20% (*42*), which corresponds to the beginning of the development of a dry region. In such a simple medium, this effect results from the fact that, below some saturation, due to the thinning of liquid films, the permeability of the liquid network is very low. Then, the liquid has not enough time left to reach the free surface under the action of capillarity to refill the water extracted by evaporation. Thus, in our case, the fact that the free water entirely disappears while the drying rate remains constant further confirms that its transport toward the free surface does not rely on a continuous liquid network.

To sum up, for slow drying, we have a porous material in which there is a noncapillary driving force tending to continuously homogenize the water distribution, without continuity of the liquid network and up to the very last volumes of free water. This means that the free water is extracted from the inner sample by another means than through a liquid flow up to the free surface. This transport through the structure could occur in the form of vapor, but the corresponding transport is rather limited. Indeed, mass flux resulting from vapor diffusion through the structure is *J*_v_ = −ρ_0_*D_v_*∇*n*, in which ρ_0_ = 0.02 kg m^−3^ is the saturation vapor density at our ambient temperature (22.5°C), *D_v_* is the diffusion coefficient, and *n* is the RH. *D_v_* may be written χ*D*_0_ in which *D*_0_ = 2.7 × 10^−5^ m^2^ s^−1^ is the coefficient of diffusion of vapor in air (*49*) and χ is a factor substantially smaller than 1 taking into account the porosity associated with the tracheid network, the fraction of free water in this network, and its tortuosity. As long as there is free water in the sample, the RH is close to 100% so that, in the absence of temperature gradient (see Materials and Methods), the maximum gradient of vapor concentration is of the order or smaller than, say 1%, over a sample length of 1 cm. Thus, the vapor mass flux is substantially lower than ρ_0_*D*_0_ ≈ 5.4 × 10^−7^ kg m^−2^ s^−1^, a value that itself is already much smaller than the macroscopic mass flux observed for this slow drying (see Fig. 3C), i.e., 2 × 10^−5^ kg m^−2^ s^−1^.

## Bound water as vector of transport of water

Under these conditions, the only possibility is that bound water be the vector of transport of free water. The free water is extracted from tracheids by absorption in the cell walls and then transported toward the free surface in the form of bound water. This process lasts as long as there is some free water in the sample. Under these conditions, the homogeneity of the free water concentration throughout the sample is likely maintained by the equilibrium of the chemical potential of bound water in the sample.

Note that this extraction of free water implies that air replaces the liquid volumes inside the structure. Since water can penetrate the structure when the sample is put in contact with it, there likely exists a continuous network of connections between tracheids, relying on open pits and micro- or nanopores (*47*, *50*), to allow for the air to flow out when the liquid arrives. The passage of an air-liquid interface through certain small openings requires very large pressure drop. However, such a connected structure is not essential to explain our observations. It was shown that in a drying hygroscopic material, the chemical potential gradient can easily induce large pressure difference, for example, leading to cavitation (i.e., vapor bubble formation) in close cavities initially filled with water inside a hydrogel submitted to a dry air flux (*51*–*54*), a material which may be considered as a model wood (*52*). This suggests that whatever the connectivity of the porous network, the driving force resulting from bound water is sufficiently powerful to extract the free water from any point. This is confirmed by previous observations of water progressively extracted from poplar fibers (*1*), which are pores with only tiny connections between each other or with vessels.

Last, as soon as there is no more free water, the bound water concentration starts to decrease first from the free surface of the sample, so that the RH conditions along the sample free surface start to substantially change and the drying rate decreases. In that stage, the relaxation time substantially decreases (see Fig. 3A). This means that, as the bound water disappears from the structure, the mobility of the remaining bound water decreases. We can infer that the molecules of the solid structure get closer, which appears qualitatively consistent with the lower apparent volume of the sample observed for decreasing bound water content (*27*).

### Strong air flux

#### Observations

For a sufficiently strong air flux tending to impose a higher drying rate, the major difference in the *T*_2_ distribution evolution is that the amplitudes of the free and bound water peaks decrease simultaneously from the beginning of the test (see Fig. 3B). The free water disappears more rapidly than the bound water, which goes on disappearing in the next stage (i.e., when there is no more free water). The relaxation time of free water nevertheless again remains constant in the first stage, while the relaxation time of bound water decreases. The simultaneous disappearance of free and bound water in the initial stage is now associated with a decreasing drying rate (see Fig. 3D) and inhomogeneous 1D free water profiles (see Fig. 3F): Rapidly, after the beginning of the test, a region without free water starts to grow from the sample’s free surface. Last, the 1D profiles keep on evolving with the growth of this apparently dry region and a simultaneous decrease in water content in the region still containing free water.

#### Analysis

The major features of a fast drying test are as follows: (i) a drying rate decreasing all along the process, (ii) the simultaneous decrease of free and bound water content in time, and (iii) the development of a region without free water growing from the free surface of the sample. This contrasts with the slow drying case for which the low rate of evaporation along the free surface lets sufficient time to water to level throughout the medium. Here, a decreasing drying rate is generally associated with an imposed evaporation, which is too fast to let sufficient water to be transported to the free surface to replace the extracted fraction. Here, the specificity with respect to simple porous media is that the “dry” region still contains bound water ensuring the transport of free water from the region below. A gradient of bound water content probably develops in this region, which explains the progressive decrease of bound water fraction from the test beginning (see Fig. 3D).

For an air flux intermediate between the two above cases, we observe initially a constant drying rate period during which only free water is extracted from the sample, and then a period of decreasing drying rate during which bound water starts to be extracted while there is still some free water. This leads to a scheme of drying rate versus time qualitatively similar to that shown in (*1*) for poplar (hardwood), whose structure differs from that of Douglas fir (softwood) while also being a hygroscopic cellular structure: As the air flux is increased, the initial constant rate period shortens. Considering the resolution of the 1D profiles with our spectrometer and the fact that we only have information on free water (see Materials and Methods), we do not attempt to detail further this intermediate case. Instead, with the help of MRI, we will focus on a drying situation that will appear qualitatively similar to the above strong air flux case, and we will follow in details both the bound water and free water distributions in time to understand the mechanisms of wood drying.

### MRI data reveal a two-step diffusion process with moving boundary

**Fig. 4.**
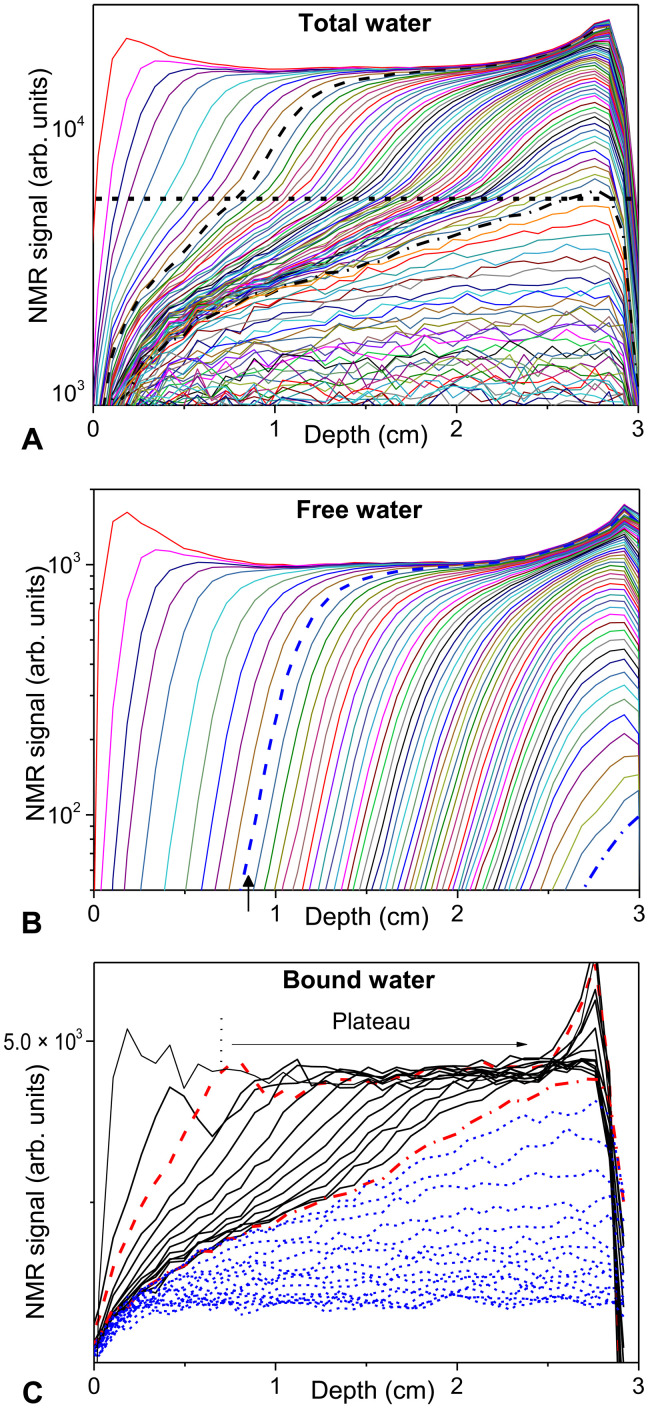
Free and bound water distributions in time. 1D water distributions in time (from top to bottom) during drying of a 3-cm-long wood sample. The dry air flux is imposed along the free surface of the sample situated at the depth zero. (**A**) Total water measured from the SPI sequence (see the main text); first five profiles every 81 min, then every 162 min up to the dash-dotted profile, and afterward every 324 min. The horizontal dotted line marks the apparent transition between two regions of transport in the sample (see text). (**B**) Free water measured from the ME sequence at same times. (**C**) Bound water as deduced by subtraction of free water from total water distributions (see text); first profile at initial time, next profile at 11 hours and 20 min, and then every 648 min. The profiles are drawn as continuous lines when some free water is still present in the sample and as short-dashed lines when there is only bound water. The dashed and dash-dotted lines in all graphs correspond to the distributions of the different water types at, respectively, 22 and 155 hours. In the latter case, this corresponds to the last profiles for which free water is still present.

Let us look at the evolution of the total water distribution in time as obtained from the single-point imaging (SPI) sequence, which has the interest to be equally sensitive to both bound and free water (see Materials and Methods) (see Fig. 4A). Globally, the profile evolution in time resembles a diffusion process: There is first some front progressing toward the sample end; when it has reached this end, the profile level progressively decreases. However, there also exists in all the profiles an inflection point around some signal level corresponding to the position of the horizontal line in Fig. 4A. This suggests that, for a water content associated with the upper region of the NMR signal (i.e., above the horizontal line), the transport dynamics differs from that in the lower region (i.e., below the horizontal line). Since there are a priori only two types of water in the material, the most natural explanation is that the upper region corresponds to free water, while the lower region corresponds to bound water.

To test this assumption, let us first look specifically at the free water distribution in time as it could be obtained from an independent measurement with the help of another imaging NMR sequence [multiecho (ME)] (see Materials and Methods) during the same drying test (see Fig. 4B). The free water profiles have two essential characteristics: They progress toward the sample bottom in the form of a front leaving a region without free water behind it, and the amount of free water beyond the front also simultaneously decreases. We take the transition to the final stage (dashed lines in Fig. 4, A and B)—when only bound water remains—as the moment when any local NMR signal of free water along the 1D profile is of the order of the noise or negligible with respect to its initial value. At this transition, there remains a substantial amount of water in the sample, as appears in the total water distributions (see Fig. 4A).

To have a complete view of the mechanisms, we need to look at the bound water distribution in time. However, the distributions for total water and for free water cannot be directly subtracted to get the bound water distribution as the signal intensity is not calibrated in the same way for two independent sequences. It would be possible to calibrate these signals from direct measurements of the total water mass and the bound water amount from sorption tests. Nevertheless, to determine the coefficient to apply on the NMR signal of free water profile so that it becomes consistent with the total water signal (i.e., the same value represents the same water mass), we prefer to use a more straightforward approach, which only relies on the analysis of the whole MRI dataset in a consistent way. To that aim, we use the fact that for the first total water profile, i.e., in the equilibrium state reached after a long imbibition process, the bound water, if it could be measured, should be uniform (for a homogeneous sample). This criterion is used to find the proper coefficient to apply on the NMR signal of free water. We then proceed to the subtraction between the first total water profile and the first free water profile signal, with a coefficient (here equal to 12.4) on the NMR signal in the latter case, allowing to get a horizontal line after this subtraction, i.e., a uniform bound water profile. This coefficient is then used (i.e., multiplied by the free water signal) to determine the successive effective bound water profiles in time, by subtracting the free water profiles from total water profiles at same time.

**Fig. 5.**
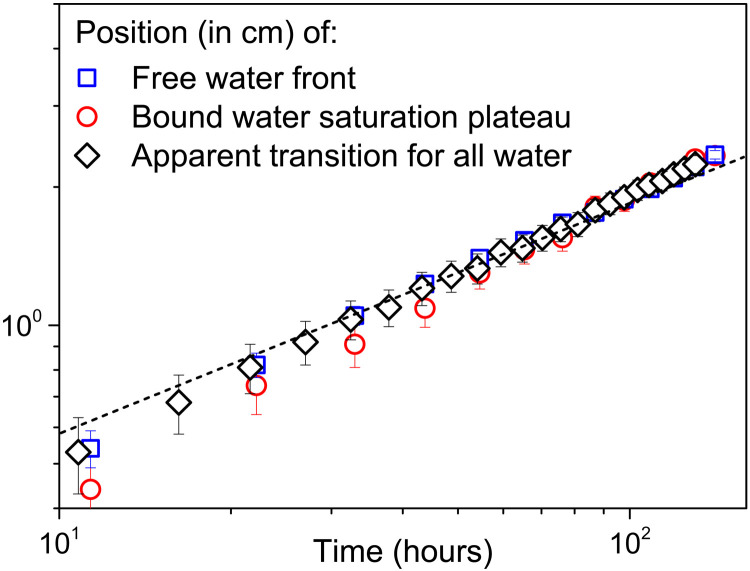
Position of the bound/free water interface in time. Drying test with 3-cm-long sample. Position (*X*) of either the beginning of the front of free water as determined from data in Fig. 4B or the beginning of the bound water plateau as determined from data in Fig. 4C and of the point of transition between the two regions in the distribution of all water as determined from Fig. 4A. The dashed line corresponds to the model $X=k\sqrt{t}$ with *k* ≈ 3 × 10^−5^ m s^−1/2^.

From these data, we can see that there is a bound water plateau beyond some depth (see Fig. 4C), which corresponds to bound water saturation. At each instant, this depth appears to be very close to the distance at which the free water profile starts and finally to the point of apparent transition between the two regions for total water (see Fig. 5). This means that we can find free water only in the region saturated with bound water. In the other region, i.e., without free water, the bound water distribution exhibits a gradient. Last, when there is no more free water in the sample, the successive bound water distributions might again be seen as globally resulting from a diffusion process from the sample surface. Note that at the end of this process, there remains a slight uniform amount of bound water (see Fig. 4C), which can be extracted only by placing the sample in an oven. A possible explanation is that the air flux is not perfectly dry and thus does not allow to induce a thorough drying.

**Fig. 6.**
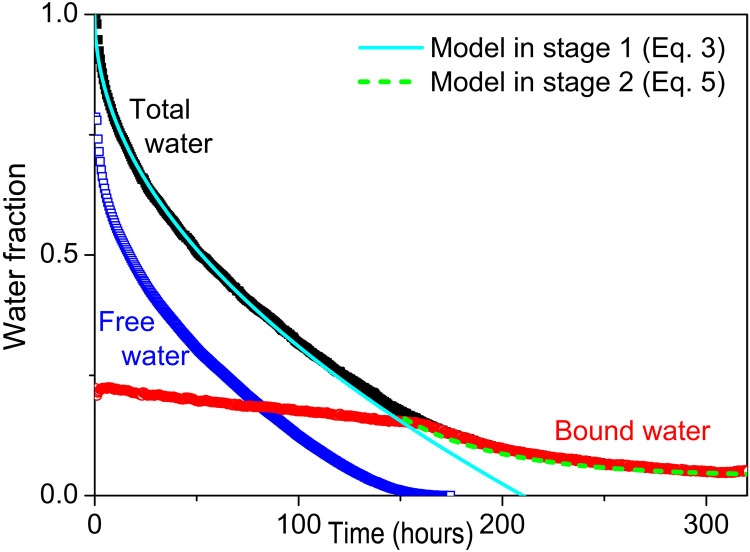
Bound/free water evolution in time. Drying test with a 3-cm sample. Evolution of each water type as a function of time, as deduced from the integration of the NMR signal in the spatial distributions (see Fig. 4) and expressed as the fraction of water mass with respect to the initial total water mass. The continuous and dashed lines correspond to the model presented in the main text. Note that the total water fraction corresponds to the saturation as defined in the text (beginning of Discussion).

Last, we can look at this experiment with the same approach as above, i.e., by distinguishing the evolution of the different types of water in the medium. In that aim, we compute the relative fractions of each water type by integrating the NMR signal distributions obtained above (taking into account the proper coefficient for free water) and dividing by the integral of the NMR profile for all water at the initial time (see Fig. 6). We can thus observe a continuous decrease of the drying rate in time, which clearly situates this experiment in the regime of strong air flux as defined above. The evolutions of bound and free water also exhibit the characteristics observed in this regime: The bound and free water fractions decrease simultaneously during a first period, and then the bound water decrease is faster when there is no more free water.

## DISCUSSION

### Simple model for bound and free water transport in wood

**Fig. 7.**
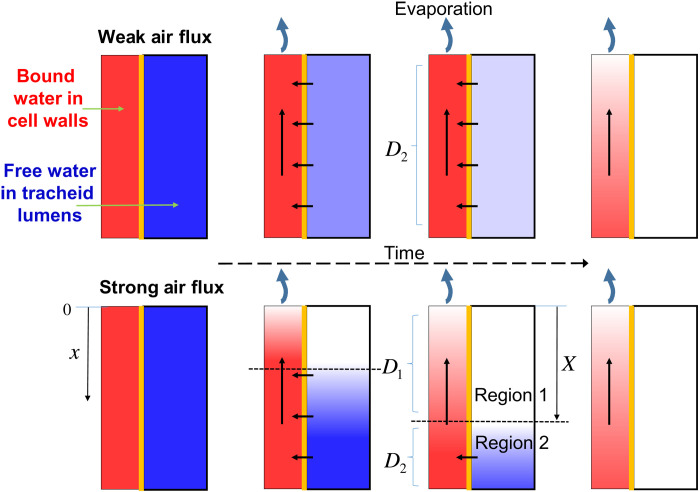
Principle of the two-step diffusion process in drying wood in the longitudinal direction. The water extraction takes place only from the upper surface. A lighter color (red or blue) indicates a lower concentration of the corresponding water type (respectively bound of free). For a sufficiently weak air flux (upper schemes), the cell walls remain filled with bound water, while the free water content in tracheid lumens decreases homogeneously, and then a bound water concentration gradient develops when there is no more free water. For a sufficiently strong air flux (lower schemes), a bound water concentration gradient develops from the free surface, in a region without free water, and extends in depth in the sample.

Let us come back to the evolution of the total water distribution (see Fig. 4A). Our analysis shows that it is essentially composed of a region 1 containing only bound water and growing from the sample free surface and a region 2 situated below the latter and in which bound water and free water coexist. The free water is transported, essentially in the form of bound water, from region 2 toward the interface between these two regions and then transported through region 1 toward the free surface still in the form of bound water. Under these conditions, we can suggest that the drying may be described essentially by two processes (see Fig. 7): a diffusion of bound water in the upper region (region 1) without free water and a diffusion of water in the lower region with bound and free water (region 2), with a different coefficient of diffusion for each region. In such a case, the interface between these two domains (i.e., respectively below and above a critical water content value) will move as the diffusion progresses, i.e., as the water content decreases in the sample, leading to a growing region of bound water alone. Obviously, this description, on the basis of two diffusion processes, each with a single (but different) diffusion coefficient, is a simplification as we can expect that the diffusion coefficient also somewhat varies with the water content in each region. Specifically, it is generally considered that the diffusion coefficient of bound water varies with the water content (*55*).

Let us consider that this drying takes the form of a transport of water by diffusion. In this context, we describe the water content via the “saturation” (ψ). Usually, for porous media, the saturation is defined as the liquid to the porous volume ratio. However, such a definition is problematic for hygroscopic materials, which can absorb water in regions initially not filled with air. In this specific context, we can define the saturation as the ratio of the current water mass to the maximum water mass value that the sample can contain. Alternatively, for a drying process starting from a uniform water content distribution, we can define it as the ratio of current water content to the initial one (MC_0_). Here, we follow the latter approach: ψ = MC/MC_0_, in which here MC is a local MC. The following developments will show that the process can be relevantly described through this unique variable. An interesting aspect of this representation is that since we are dealing with diffusion phenomena, the qualitative characteristics of the dynamics are independent of the initial MC in the sample. Thus, similar evolutions in terms of ψ should be observed whatever MC_0_.

According to the above observations and conclusions, we assume that the medium exhibits two different diffusion coefficients depending on the value of the saturation ψ: *D*_1_ for ψ < ψ*_c_* (region 1), *D*_2_ for ψ > ψ*_c_* (region 2). Here, ψ*_c_* is the critical saturation beyond which free water exists, while for ψ < ψ*_c_* we only have bound water. Here, it is estimated as the ratio of NMR signal in the bound water plateau (see Fig. 4C) to the total NMR signal in the same region (from Fig. 4A data), and we find ψ*_c_* ≈ 0.26. Note that ψ*_c_* depends on MC_0_ and the FSP: ψ*_c_* = FSP/MC_0_.

The above approach, which assumes a single initial MC value, implicitly means that we do not consider the regions situated at the edges of the sample where the initial MC is larger than elsewhere (see Fig. 4A). In the following, depending on the type of data analysis, we will be able to either damp the effect of such regions by some rescaling with the initial profile or remove the corresponding parts of the profiles without substantial impact on our conclusions. Considering the whole consistency of the analysis and modeling, this approximation appears to be relevant. This approximation has nevertheless one visible impact on the apparent bound water fraction obtained by integrating the NMR signal, which is consequently slightly smaller (i.e., 0.23 instead of 0.26) (see Fig. 6) than ψ*_c_* as this integral is taken over the whole distribution.

### Stage of coexistence of bound and free water

To solve the diffusion problem, we take the simple boundary condition ψ(*x* = 0) = 0, which apparently constitutes a good approximation of our MRI data (see Fig. 4A). After a few hours of drying, when the water profiles exhibit two distinct regions the saturation at the approach of the sample free surface tends to zero and remains at this level all along the drying process. We also assume that the medium is semi-infinite (no right boundary in the region ), and we take the initial condition as ψ(*x* > 0, *t* = 0) = 1, where *t* is the time.

The solution of this diffusion problem [see (*56*)] may be expressed as follows

$$\psi =A\text{erf}(x/2\sqrt{D_{1}t})\;\text{for}\;0<x<X$$ (1a)

$$\psi =1-B(1-\text{erf}(x/2\sqrt{D_{2}t}))\;\text{for}\;X<x$$ (1b)

in which *A* and *B* are two parameters and *X* is the position of the interface between the two regions (see Fig. 7), which expresses as $X=k\sqrt{t}$ (as part of the mathematical solution of the problem), where *k* is a parameter. Taking into account the saturation continuity at the interface: ψ(*X*_−_) = ψ(*X*_+_) = ψ*_c_*, the above system may be rewritten as

$$\psi =\psi_{c}\frac{\text{erf}(\alpha u)}{\text{erf}(\alpha )}\;\text{for}\;0<u<1$$ (2a)

$$\psi =1-(1-\psi_{c})\frac{\left(1-\text{erf}(\beta u)\right)}{1-\text{erf}(\beta )}\;\text{for}\;1<u$$ (2b)

with *u* = *x*/*X*, $\alpha =k/2\sqrt{D_{1}}$, $\beta =k/2\sqrt{D_{2}}$.

**Fig. 8.**
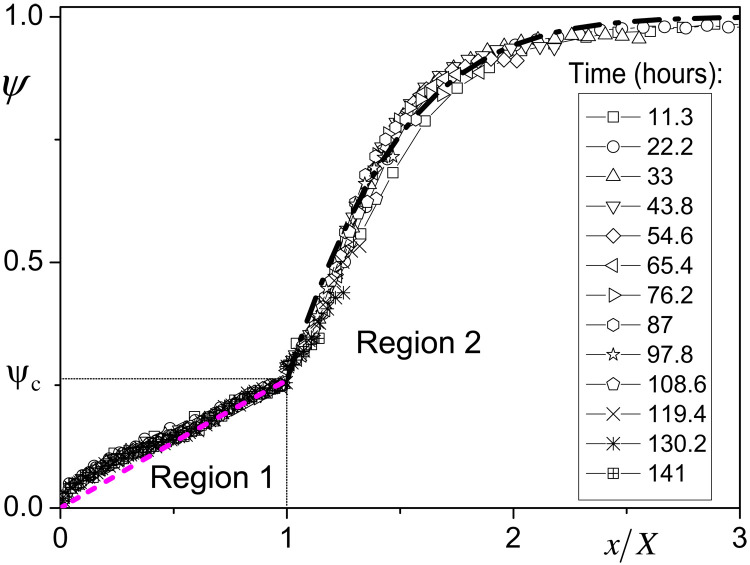
Rescaled saturation profiles and comparison with model. Saturation profiles in time for the MRI experiment for bound water and all water as a function of the position along the sample axis rescaled by the value of *X* respectively associated with the beginning of the bound water plateau or the front of free water. The bound water saturation profiles were obtained by rescaling the NMR signal in Fig. 4C data by its central plateau value for all water (see Fig. 4A). The total water saturation profiles here correspond to the free water profiles (see Fig. 4B) properly rescaled and to which the saturation value associated with the bound water fraction at saturation (ψ*_c_*) has been added. Note that here, the free water profiles have been beforehand rescaled by the first one; this allows to artificially get an initial uniform saturation despite the heterogeneities at the edges (see Fig. 4B) but does not affect the relevance of the analysis as the main inhomogeneity (i.e., the peak) is situated around the sample bottom, i.e., at the right end of each curve. The dashed and dash-dotted lines correspond to the theory for each region, i.e., respectively Eqs. 2a and Eqs. 2b.

We can first check that the saturation profiles follow the general above shape. Since the position of the transition between the two regions observed from the total water signal (see Fig. 4A) cannot be precisely determined, we do not directly use these data. Instead, we use the data obtained specifically for free and bound water (see Fig. 4, B and C) and the corresponding positions of the end or the beginning of these regions (see Fig. 5), referred to as *X* in both cases, to rescale the distance *x*. This rescaling allows getting master curves for the saturation in each of the regions [see Fig. 8), as expected from the form of the model, i.e., a dependence on time and distance through *x*/*X* only (Eq. 2)].

We can then determine the value of β by a best fit procedure (in the range 1 to 3 for *u*) of the model to this master curve in region 2. We get β ≈ 0.83. Note that this is the only step of fitting on the model, all other parameters will be deduced from this value and a direct measurement of *k* (see below).

Then, we write the condition of flux continuity at the interface: *D*_2_(∂ψ/∂*x*)_*X*+_ = *D*_1_(∂ψ/∂*x*)_*X*_−__. Taking into account the expressions (Eq. 2), this condition writes: βψ*_c_* exp (β^2^)(1 − erf (β)) = α(1 − ψ*_c_*) exp (α^2^) erf (α), from which we deduce α ≈ 0.34. The model (i.e., Eq. 2a) with this value is in agreement with data in region 1 (see Fig. 8), but considering the limited range of *u* (i.e., 0 to 1) in which we appreciate this agreement and the local slope variations of the curves in this range, this description with a single diffusion coefficient in this region may be considered as a rough global approximation.

At last, we can see (see Fig. 5) that the position of the interface *X* between the two domains effectively varies with the square root of time, as predicted by the theory, and we have *k* ≈ 3 × 10^−5^ m s^−1/2^. We deduce *D*_1_ = *k*^2^/4α^2^ ≈ 1.95 × 10^−9^ m^2^ s^−1^ and *D*_2_ = *k*^2^/4β^2^ ≈ 3.3 × 10^−10^ m^2^ s^−1^ .

Note that this apparent excellent agreement between the model predictions and the data is obtained despite the fact that the boundary conditions do not correspond exactly to the theory, which assumes a semi-infinite medium. This is partly due to the fact that the final profiles in this stage (when free water is still present), which are certainly affected by the sample end, play a minor role in the plot of all rescaled data since the corresponding points are pushed toward the region *u* = 1 due to this procedure. It is also remarkable that this agreement is also obtained despite the use of unique diffusion coefficients in each region, but we will see later that some discrepancy appears when data in region 1 are looked at in more details.

We can also remark that the value found for *D*_1_ corresponds to the typical ones found for the longitudinal diffusion coefficient of bound water in pine from standard macroscopic measurements (*55*). As far as we know, the value of the diffusion coefficient in the region containing both bound and free water has not been measured so far. The possible physical origin of this transport process and the corresponding diffusion coefficient value are discussed below.

From this model, we can also easily derive the theoretical expression for the drying rate. The drying rate is defined as the water mass loss per unit time and unit surface exposed to air flux, i.e., *J* = − (*m*/*S*)dMC/d*t* where *S* is the area of the sample surface. Using $\bar{\psi }$ the average saturation (over the whole sample), we deduce the drying rate in terms of volume loss per unit time and unit cross-sectional area, i.e., $V=-(m{\text{MC}}_{0}/\rho S)d\bar{\psi }/dt$. As soon as there is a region 1, i.e., no more free water around the sample free surface, we have a flux of saturation *D*_1_(∂ψ/∂*x*)_*x* = 0_ leaving the sample. From Eq. 2a, this corresponds to a volume loss per unit time and unit cross-sectional area $\nu D_{1}{\left(\partial \psi /\partial x\right)}_{x=0}=\nu (\psi_{c}/\text{erf}\alpha )\sqrt{D_{1}/\pi t}$ in which *v* = *m*MC_0_/*SH*ρ is the initial water volume fraction, where *H* is the sample thickness (i.e., height along the direction *L*). Equating this expression with the previous one, we find after integration

$$\bar{\psi }=1-\frac{\psi_{c}}{\text{erf}\alpha }\frac{2}{H}\sqrt{D_{1}t/\pi }$$ (3)

which gives the variation of the average saturation in time. Note that this expression is valid only as long as there exists some free water in the system. This expression, in which we use the value for *D*_1_ deduced from the saturation distribution evolutions, appears to almost exactly predict the observed experimental variation of the average saturation in time as long as there is some free water in the sample (see Fig. 6).

### Stage of bound water alone

Let us now check that in the next stage, i.e., when there is only bound water, the evolution of saturation distribution is consistent with the simple diffusion mechanism assumed above. For the sake of simplicity, to deal with this stage in details, we rescale data by considering that the equilibrium state under the imposed air flux corresponds to the apparent asymptotic plateau observed for ψ ≈ 0.05 (see Fig. 5), and we follow the reduced saturation ξ = (ψ − 0.05)/(ψ*_c_* − 0.05). Thus, ξ varies between 0 and 1 and follows the same diffusion equation as ψ.

We look at the solution of the diffusion problem assuming that ξ(*x* = 0) = 0 all along the process, which appears to be in agreement with the observations. Moreover, we assume that at some initial fictive time *t*_0_, ξ(*x* ) = 1, i.e., uniform saturation ψ = ψ*_c_* (only bound water). Under these conditions, the solution of the diffusion problem for ξ(*x, t*′) with *t*′ = *t* − *t*_0_ writes (*56*)

$$\xi (x,t\prime )=\frac{4}{\pi }\sum_{i=0}^{\infty }\frac{{\left(-1\right)}^{i}}{2i+1}[\begin{array}{c} \text{exp}\{-D_{1}{\left(2i+1\right)}^{2}\pi^{2}t\prime /4H^{2}\}\\ \times \text{cos}\frac{\left(2i+1)\pi (H-x\right)}{2H} \end{array}]$$ (4)

**Fig. 9.**
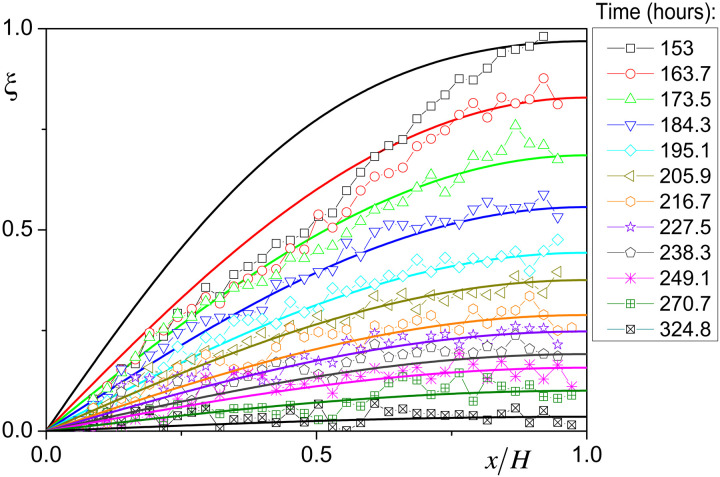
Reduced bound water saturation distributions in time (*t*) when bound water only is present in the sample. The continuous lines correspond to the model predictions (Eq. 4) at the same times.

Note that for the comparison with data, we simply estimate this origin of time *t*_0_ by a direct fit of the theoretical prediction with the first saturation profile observed in this stage (see Fig. 9). Here, we find *t*_0_ ≈ 141 hours, which implies that the time *t*′ for which we have the first experimental profile (*t* ≈ 153 hours) in this stage is 12 hours.

The set of distribution profiles predicted by the model under these conditions appears to be in excellent agreement with data for ξ < 0.7, i.e., for *t* > 173 hours (see Fig. 9). The only discrepancy affects the profiles reaching the region of larger values (i.e., for *t* < 173 hours). In that case, the shape of the profiles substantially differs from the theoretical ones: It seems that the profiles include two successive concave parts with a junction point around ξ = 0.5. Actually, such a shape looks similar to that observed for the master curve of the bound water distribution in the previous stage (coexistence of bound and free water) (see Fig. 9), which confirms that there is at first sight two domains of saturation with different diffusion properties. These rescaled profiles correspond to the bound water distribution under various situations of advancement of the interface between regions 1 and 2. Thus, the specific shape of the master curve cannot be associated with some heterogeneity of the sample structure, it truly concerns different diffusion characteristics of bound water only depending on the saturation value. Further analysis of such data could provide some insight in the variation of the diffusion coefficient value as a function of the saturation reported in literature from macroscopic data (*55*).

However, it is worth emphasizing that, except for this particular shape, our global description with a single diffusion coefficient for the region or stage with bound water alone allows predicting well all the dynamics and shapes of the water distributions in time in any case (see Figs. 8 and 9), as well as the average saturation versus time curve in the stage of bound and free water coexistence (see Fig. 6). We can now check the ability of this approach with a single diffusion coefficient to describe the macroscopic variation of the saturation in time in the stage of bound water alone. In that case, the theory predicts for the average reduced saturation

$$\bar{\xi }=\sum_{i=0}^{\infty }\frac{8}{{\left(2i+1\right)}^{2}\pi^{2}}\text{exp}\{-D_{1}{\left(2i+1\right)}^{2}\pi^{2}t\prime /4H^{2}\}$$ (5)

from which we deduce $\bar{\psi }(t)$ in the stage of bound water alone, which also appears to be in good agreement with data (see Fig. 6).

Last, this simple model is able to describe phenomenologically the data very well, keeping in mind that the description of the processes is likely rough particularly in region 1 (no free water). An improved approach should take into account the detailed variation of the diffusion coefficients as a function of saturation in each domain.

### Application to any drying conditions

The above analysis provides a detailed view of the internal processes during a drying under particular conditions, which actually gives us the full phenomenological characteristics of water transport inside the medium. Using these properties, we can now deduce the general drying characteristics under any conditions.

Let us consider a convective drying process under any conditions of air flux along the free surface. We first need to describe the boundary conditions and how they affect the drying rate. The situation is not as simple as one might assume; the drying rate is not simply and directly imposed by these boundary conditions, it is the result of some equilibrium between the intensity of the external dry air flux and the rate at which the water can be transported through the medium toward the sample free surface.

Drying occurs as a result of the unbalance between the internal humidity conditions (i.e., inside the sample) and the external conditions. Here, this unbalance is maintained by the dry air flux imposed above the sample surface, while the RH inside the sample is larger than 0 as long as it contains some water. Since the air flux can hardly penetrate the sample, which is a finely divided dead end network, the air essentially steadily flows along this surface and is generally almost uniform at a short distance from the surface. For such an air flow along a wet surface, it may be shown by solving the advection-diffusion equation that from each point of the surface the gradient of RH in the air above, i.e., along the direction normal to the free surface, is almost constant, so that all occurs as if the vapor was diffusing freely from this point of the sample surface over a distance δ where it reaches the region where a RH *n*_0_ is imposed. Actually, the value of δ depends somewhat on the position along the sample surface, on the surface shape, the size and roughness, and, more critically, on the upstream air flux characteristics. Then, we can consider as a first approximation (*57*) that a single average value δ, associated with the specific conditions, allows to represent the whole process of vapor transport above some given sample surface, so that the mean vapor flux (*J*_0_) in the direction normal to the surface, which is also the drying rate (water mass flux per unit surface), may finally be written

$$J_{0}=-\frac{m}{S}\frac{d\text{MC}}{\mathrm{dt}}=\rho_{0}D_{0}\nabla n$$ (6)

with ∇*n* = (*n_i_* – *n*_0_)/δ where *n*_0_ = 0 is the RH at the distance δ (assuming a dry air flow) and *n_i_* is the (average) RH along the sample surface. For a drying experiment starting with a sufficiently wet sample, we expect to find a high density of wet patches along the free surface so that we can reasonably assume that we are in the conditions described by Schlünder (*58*) and Suzuki and Maeda (*59*) and later fully quantified (*43*), for which *n_i_* ≈ 1 at a very short distance from the sample surface. Then, δ can be directly determined from a measure of the initial drying rate of the sample since in that case *J*_0_ = ρ_0_*D*_0_/δ. This situation (i.e., *n_i_* ≈ 1) is equivalent to that obtained along the free surface of a pure liquid water layer, which implies that alternatively, if the sample roughness has a negligible impact, then δ might be estimated from a test with the sample replaced by a water layer of same shape. This further illustrates the true nature of δ, which is solely imposed by the air flux and on which the internal material properties have no impact. This parameter characterizes the boundary condition; for our tests, δ ranged from 2.3 to 40 mm.

In the next step, i.e., just after the initial stage, the current drying rate results from the coupling between the imposed air flux and the ability of water in any state to be transported through the material. This coupling leads to some provisional equilibrium between the vapor diffusion above the sample and the current distribution of water in the sample so that the RH along the free surface may be smaller than one, leading to a lower drying rate.

Let us now consider the convective drying of a sample whose characteristics are those above described (i.e., with two different diffusion coefficients depending on the saturation value) and with an initial uniform saturation larger than ψ*_c_*. When the drying starts, the saturation along the free surface is larger than ψ*_c_*, and as long as this remains true, a first stage takes place for which the drying is described by a simple diffusion process throughout the sample, with a unique diffusion coefficient *D*_2_. The boundary conditions are now no flux for *x* = *H* (sample bottom) and a flux *J*_0_ at the interface (*x* = 0) imposed by the air flux, which according to Eq. 6 gives ρ*D*_2_(∂ψ/∂*x*)_*x* = 0_ = ρ_0_*D*_0_*n_i_*/δ, where ρ is the liquid water density (pure water under ambient conditions). As long as there is free water in the porous sample up to the free surface, the humidity conditions around *x* = 0 can be considered to be *n_i_* ≈ 1. Here, this result directly derives from the fact that the material is saturated with bound water, so that it is at equilibrium with an ambient RH equal to 1. As a consequence, as long as ψ > ψ*_c_* up to the sample free surface, the drying rate remains constant and equals the volume loss per unit time and unit cross-sectional area deduced from the water flux at the interface ν*D*_2_(∂ψ/∂*x*)_*x* = 0_ = ρ_0_*D*_0_/ρδ. We then define the flux of saturation as

$$F_{0}=D_{2}{\left(\partial \psi /\partial x\right)}_{x=0}=\rho_{0}D_{0}/\nu \rho \delta$$ (7)

Remark that *F*_0_ provides a simple appreciation of the drying conditions imposed by the air flux: For a pure water layer, this would represent the velocity of decrease of the layer thickness. For our experiments, *F*_0_ ranges from 1.1 to 40 mm day^−1^.

Under these conditions, the diffusion problem is that of a plane sheet (of finite thickness *H*) with a constant flux of extraction *F*_0_ imposed along its surface and a given diffusion coefficient *D*_2_, whose solution expresses as (*56*)

$$1-\psi =\frac{F_{0}H}{D_{2}}[\begin{array}{c} \frac{D_{2}t}{H^{2}}+\frac{3x^{2}-H^{2}}{6H^{2}}\\ -\frac{2}{\pi^{2}}\sum_{m=1}^{\infty }\frac{{\left(-1\right)}^{m}}{m^{2}}\text{exp}(-D_{2}m^{2}\pi^{2}t/H^{2})\text{cos}\frac{m\pi x}{H} \end{array}]$$ (8)

Here, the characteristic time for emptying the sample is *T* = *H*/*F*_0_. If for this time the first term in brackets in Eq. 8 is much larger than 1, this term is dominant, and the saturation profiles are essentially horizontal over the whole test duration. This condition expresses as *D*_2_/*HF*_0_ > > 1 or, more precisely, taking into account the boundary conditions

$$\text{Dr}=\frac{\nu \rho D_{2}\delta }{H\rho_{0}D_{0}}>>1$$ (9)

On the contrary, for Dr << 1, the second term in brackets in Eq. 8 is dominant so that the profiles are again essentially parallel but now with a parabolic shape at the approach of the free surface. When the critical saturation is reached by this saturation distribution at the sample free surface (where it takes its lowest value), there is no more free water and the bound water starts to disappear, we are in a regime involving the two diffusion processes as described above. From a qualitative point of view, such a behavior appears to be fully consistent with the observations for the strong air flux and/or large sample thickness.

Last, three different drying regimes can be distinguished depending on the time spent in the constant rate period associated with a homogeneous decrease of the saturation and unique diffusion coefficient:

1) Dr >> 1 Regime A. Homogeneous free water extraction: Constant drying rate up to complete emptying of free water and uniform saturation distribution and then bound water extraction associated with a drying rate decrease;

2) Dr ≈ 1 Transitional regime: Constant drying rate with a gradient of free water during some initial period; then, diffusion with two regions, a moving interface and a decreasing drying rate; last, extraction of the remaining bound water alone;

3) Dr << 1 Regime B. Two-step diffusion: Almost immediately decreasing drying rate associated with diffusion with two regions and then extraction of the remaining bound water alone; as soon as free water has disappeared from the top layer of the sample, the water distribution profiles are those described in “Stage of coexistence of bound and free water” section above, and the average saturation is given by Eq. 3.

**Fig. 10.**
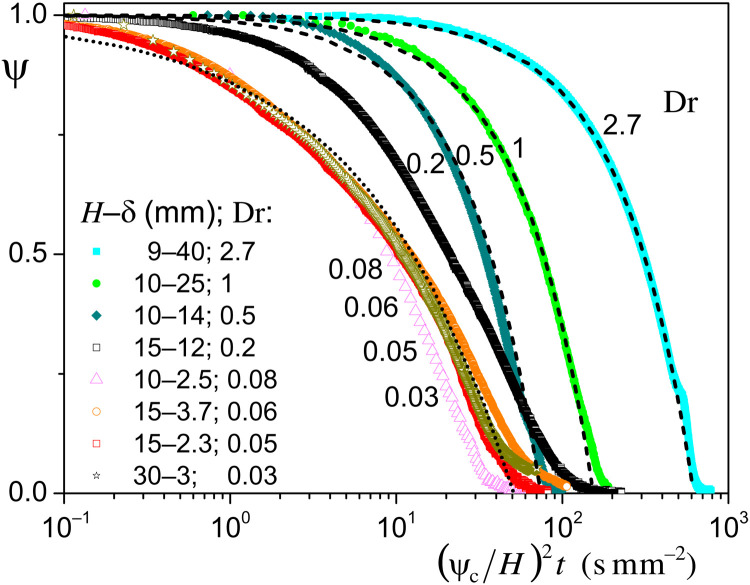
Drying curves for tests under different conditions of air flux and sample size (thickness). The value of Dr is indicated for the different curves to illustrate the ranges associated with the two main drying regimes (regimes A and B, see the main text). The dashed lines are simple models with a constant drying rate, and the dash-dotted line is the model prediction under the assumption of bound water front developing from the beginning of the test (Eq. 3).

We can check the validity of this description by looking at the drying curves (saturation versus time curves) for the above tests and a series of additional data obtained for different sample thicknesses and under different air flux intensities (see Fig. 10). To be able to relevantly compare such data, one can rescale the time by (*H*/ψ*_c_*)^2^. Under these conditions, according to Eq. 3, for small Dr, we expect in theory a master drying curve in such a representation, while for large Dr, we expect a straight drying curve with a slope −*F*_0_*H* thus proportional to 1/Dr. For Dr = 2.7 the drying curve in linear scale was shown to keep a constant slope during most of the desaturation (see Fig. 3C). Since in logarithmic scale the drying curves for Dr > 0.4 are parallel to the curve for Dr = 2.7, we deduce that in linear scale they also exhibit a constant slope over most of the time. Under these conditions, the drying curves for Dr > 0.4 are well represented by constant drying rate models and move to larger times for larger Dr values (see Fig. 10).

In contrast, for sufficiently low Dr values, the curves superimpose although Dr goes on decreasing substantially. The transition between regime A [straight drying curve (in linear scale) with a different drying rate] and regime B (master drying curve) seems to be in the range of 0.2 to 0.5 for Dr. In this range, e.g., for Dr = 0.2, the drying curve starts with a constant slope but around a (mean) saturation of 0.5 turns into the shape observed for low Dr, a change corresponding to a decrease of ψ below ψ*_c_* below the sample free surface.

### Mechanism of transport of bound water

For a variety of wood types, including different hardwoods and softwoods, internal observations of water evolution during wood drying showed that bound water appeared to be extracted along free water in some cases, while in other cases, it was extracted only when all free water has disappeared (*1*, *4*, *32*). Our findings here allow to rationalize and explain these observations: constant drying rate and full removal of free water first for slow drying or decreasing drying rate and simultaneous decrease of both water types resulting from a two-step diffusion inside the sample. This also demonstrates the generality of our main findings (as defined above) through its application to this variety of wood and wood types.

The diffusion of bound water through the structure when there is no more free water in the sample is something expected as it directly results from a gradient of the chemical potential of bound water due to a gradient of its concentration. The diffusion process in the medium when free water and bound water coexist is more unexpected. It no doubt results from a gradient of concentration of water along the sample axis, but the driving force remains to be identified. Here, we suggest an explanation based on the assumption that the partial saturation in free water is at the origin of a slight deficit of bound water in the cell walls, which induces a difference of chemical potential with respect to its value at bound water saturation.

In the region where it coexists with free water, the bound water saturation is a priori maximum, so that there is no concentration gradient allowing transport. As a consequence, under these conditions, the bound water transport is analogous to the flow of liquid through a saturated porous structure under the action of a pressure gradient.

Thus, we need some pressure gradient resulting from differences of saturation in free water and which will induce the bound water transport. We can suggest that, as soon as some fraction of the cell walls is not directly wetted by the liquid, the RH is slightly smaller than 1, which has an impact on the pressure of bound water in the cell walls around this point. Such an effect could in particular occur as a result of the concentration of salts (*60*) in the free water remaining in the cavity, which decreases the water activity. This leads to an effect similar to the capillary effects in the liquid network in a simple porous medium, but here, since the transport is ensured by bound water that remains close to saturation, there is no decrease of the permeability as long as the bound water content is close to saturation. This would in particular explain that we can fully desaturate the medium of its free water while keeping a constant drying rate, in contrast with a simple porous medium with standard capillary effects; in the latter case, the permeability decreases when the saturation decreases, which eventually induces a slowdown of the transport leading to the development of a dry front growing from the free surface.

The simplest way to represent this impact of the free water content on the RH is to write it as

n=1−a(1−s) (10)

where *a* is a coefficient such that *a* < < 1, and *s* is the specific saturation of free water (ratio of free water content to its maximum possible value). Then, we write the equality of the chemical potential between the sample top, where we have a RH *n*_0_ and a pressure *p*_0_ (ambient air), and any depth inside the sample, where we have *n* and *p*. This leads to (*51*)

$$p_{0}-p=-\frac{RT}{\Omega_{m}}\text{ln}(\frac{n_{0}}{n})$$ (11)

where Ω*_m_* = 1.8 × 10^−5^m^3^mol^−1^ is the molar volume of liquid water, and *R* = 8.31 J mol^−1^ K^−1^ is the gas constant. From Eqs. 10 and 11, we deduce the pressure gradient $\nabla p=\frac{\mathrm{aRT}}{\Omega_{m}}\nabla s$.

On another side, we can write the equivalent of a Darcy’s law for the bound water $\nabla p=-\frac{\mu }{K}V$ in which μ is the bound water viscosity, *K* is the permeability of the medium to bound water at saturation, and *V* is the flow rate per unit cross-sectional area. Note that although such a linear relationship is likely valid, as was, for example, found from molecular simulations for hydrocarbon transport (*61*), the expression of the coefficient through the ratio of these two parameters (i.e., a viscosity and a permeability) is much more questionable (*61*). We will nevertheless keep such an expression, assuming it provides reasonable orders of magnitude of the physical processes.

Considering that the pressure of bound water is equal to that in the air around we deduce

$$V=-\frac{\mathrm{aK}RT}{\mu \Omega_{m}}\nabla s$$ (12)

Since the only longitudinal transport of free water is in the form of bound water, *V* is finally the flow rate of free water per unit cross section, and Eq. 12 may be seen as a first Fick’s law for the free water, from which we naturally derive

$$\frac{\partial s}{\partial t}=-D\frac{\partial^{2}s}{\partial t^{2}}$$ (13)

with $D=\frac{\mathrm{aK}RT}{\mu \Omega_{m}}$. Thus, the free water transport can be described as a diffusion process.

Note that if we take the value of the diffusion coefficient *D*_2_ determined from our experiments and using for μ the standard viscosity of water at 25°C, then we should have $\text{aK}=\frac{\mu \Omega_{m}}{RT}D_{2}\approx 2\times 10^{-21}\;m^{2}$. If we have, for example, *a* = 0.01, this leads to a permeability of the order of 2 × 10^−19^ m^2^, which would correspond to the permeability (*R*^2^/8) for a Poiseuille flow through cylinders of radius *R* of the order of a nanometer, in consistency with the space likely allowed for water molecules inside the cell walls.

The drying characteristics observed for sufficiently large thickness and/or strong air flux likely plays a critical role on the plant behavior since:

1) it delays the sample drying;

2) it maintains the drying rate at some value whatever the air flux intensity beyond some value; for example, the time for full drying does not seem to decrease further for Dr decreasing below 0.8 (see Fig. 10);

3) as a corollary, it allows to store free water a longer time in depth;

4) the presence of bound water in the upper driest region instead of a fully dry region allows to maintain some hydraulic continuity throughout the material; and

5) this hydraulic continuity will facilitate the water recovery as soon as the material will be placed again in contact with a water source, i.e., the bound water ensures the process reversibility.

These results also definitely show that drying is controlled by bound water, which induces a sufficiently fast transport throughout the material if the evaporation rate is not too large. This suggests that there is another possibility than capillary effects to induce water climbing at large heights in trees. Although free water is finally absent over some length, bound water diffusion up to the leaves could be sufficiently powerful to ensure this climbing.

These results provide an understanding of the basic mechanisms to take into account when dealing with wood drying in practice. They also suggest to devise new models able to properly take into account the development of different regions in the sample in which the water transport characteristics differ.

## MATERIALS AND METHODS

### Material and drying setup

The materials used are wood samples collected from Douglas fir planks, which had been left drying naturally under ambient conditions. Note that tests carried out with green samples of the same species showed qualitatively similar results, but we leave a detailed comparison for future studies. The samples are cut along the wood anisotropic directions with sizes *R* × *T* × *L* = 5 mm by 5 mm by 10 mm for NMR experiments and *R* × *T* × *L* = 36 mm by 36 mm by 15 mm or 30 mm for other tests (MRI or simple weighing). All samples were taken in sapwood, and we do not distinguish whether the samples were taken in particular in early or latewood (for the small samples) as this does not appear to play a role on the transport phenomena during our drying experiments. Before the tests, the samples were immersed in water for several weeks for imbibition. The amount of water inside the structure is described through the MC. Thus, the water content in the material is equal to *m*MC. With our samples, the initial MC after imbibition ranged from 62 to 100%. Since for such materials, the maximum MC is up to 150% (for green wood), this means that the samples were initially not saturated. Actually, this is not a problem within the frame of our description of the phenomena for homogeneous initial water content, since diffusion processes only depend on the concentration, which, in our case, corresponds to the saturation. The FSP of our material, determined from sorption experiments [see (*62*)], corresponds to a MC of 22%.

The samples were covered with an impermeable tape on all sides except the bottom surface and the top surface. The tape is a Teflon film that lies in close contact with the top surface of the roughness of the wood sample, thus forming an artificial wall. Last, the resulting local structure in this region should resemble that in the bulk, i.e., with tracheids (possibly partial sized), rays, and cell walls, so that we do not expect this region to play a specific role in the observed processes.

The temperature is maintained at 22°C. For both NMR and MRI experiments, the samples are surrounded by a cryostat (situated around the coils), which tends to maintain a constant temperature, and the whole setup (equipment and sample) is placed in a room with a system regulating the same temperature. Last, the samples are subjected to a (vertical) dry air flux normal to their free surface and at the same temperature. Under these conditions and considering the long duration of the experiments (say more than a few tens of hours for small samples and several hundreds of hours for large samples), temperature gradients in the sample were negligible. Some tests were also carried out by imposing a horizontal dry air flux along the free surface; in that case, only the weight of the sample was followed in time, from which we could deduce the water loss.

Despite the anisotropic structure of the system, we can consider that the sample is homogeneous as long as we look at its properties through 1D distribution measurements, i.e., through quantities corresponding to the average of some local quantities over cross-sectional layers. Considering the sample size, each such cross section goes through a great number (typically of the order of one thousand for NMR 1D profiles and several times more for the MRI tests) of tracheids and thus through various locations along tracheids. In addition, note that we can neglect the sample shrinkage along the longitudinal direction as it is not larger than 1%. A greater shrinkage occurs in the other directions but does not affect the dynamics, which we strictly observe in the longitudinal direction.

### NMR and MRI

#### Experimental procedures

NMR allows to directly access the movement of water molecules and specific interactions of water with its environment (e.g., adsorption, proton lability, presence of paramagnetic elements, etc.) through its relaxation times. Here, we use NMR and MRI techniques allowing to follow free and bound water.

For ^1^H NMR measurements on small wood samples, we used a Bruker NMR minispec mq20, 0.5 T, 20 MHz. We measure the transverse relaxation time *T*_2_ using a Carr-Purcell-Meiboom-Gill (CPMG) sequence (*63*) with the following main parameters (adapted in order for all populations to reach magnetization equilibrium): number of scans: 64; echo time: 0.4 ms (i.e., less than the minimum *T*_2_ for bound water); number of echoes: 4000; and repetition time: 8 s, for a total measurement duration (for one *T*_2_ distribution) of 6 min, which is much lower than the total duration of the drying test. We lastly get an apparent statistical distribution of *T*_2_, expressed in terms of signal intensity associated with each possible value of *T*_2_. The *T*_2_ distributions thus obtained exhibits characteristic peaks of different magnitudes at different relaxation times associated with each type of water, in consistency with previous observations (*1*). Note that the Laplace inversion still suffers from some important drawbacks and artifacts (*64*, *65*), and the exact shape of this distribution somewhat depends on the processing protocol (*66*). However, for given NMR sequence parameters, the relative amplitude of the peaks, as well as the evolution of their shape over time, is robust data (*64*).

During the same tests, it was also possible to get, with spin-echo sequence, 1D NMR profiles of free water with this NMR system equipped with a 4 T/m vertical pulsed gradient unit. The technique was described elsewhere [see (*1*)]. These 1D profiles obtained by Fourier transform represent the spatial distribution of water along the longitudinal axis *z*, each data point corresponding to the free water content in a cross-sectional layer of thickness equal to the spatial resolution. The main parameters used are as follows: number of scans: 64; echo time: 7.2 ms; field of view: 20 mm; number of pixels: 128; repetition time: 8 s, for a total measurement duration of 11 min. The minimum echo time that can be imposed results from technical limitations of the apparatus. Because of this echo time substantially larger than the relaxation time of bound water, along with additional effects related to the magnetic field gradient, here only free water is detected by this technique [see also (*1*) for more details].

For ^1^H MRI measurements, larger samples were inserted in a 24/80 DBX 0.5-T ^1^H MRI spectrometer by Bruker (20-cm open diameter in the sample area), operating at 20 MHz. First, we look at the evolution of the spatial water distribution in time from quantitative 1D profiles of the liquid content along the longitudinal axis *z*. These measures were obtained by means of the SPI sequence (*67*, *68*). The parameters of the MRI sequence used are as follows: number of scans: 256; signal collecting time: 400 μs; field of view: 50 mm; number of pixels: 64; flip angle: 15°; repetition time: 100 ms, for a total measurement duration of 27 min. These parameter values were adjusted to be sensitive to all water types in wood while keeping a high signal to noise ratio. It was checked through preliminary tests with copper sulfate solutions with adjusted relaxation times that, with this sequence and our parameters, we get the same NMR signal amplitude for the same mass of water, whatever bound or free. The data analysis method was described in a previous study (*69*). The advantage of using this sequence is the possibility to study materials with very short transverse relaxation time *T*_2_, i.e., somewhat shorter than typically 1 ms, in contrast with more classical spin-echo or CPMG sequences. As a consequence, here, this sequence allows us to detect all water, including bound and free water.

Second, with the same MRI system, we measure the *T*_2_ distribution with a ME sequence using the following parameters: number of scans: 128; echo time: 7.5 ms; number of echoes: 16; field of view: 50 mm; number of pixels: 64; repetition time: 1 s for a total measurement duration of 2 min. The Fourier transform of each echo allows obtaining a profile, which, for each pixel, is fitted by an exponential function *A* exp (− *bt*) where *t* is the number of echoes multiplied by the echo time and *A* is the amplitude associated with the free water content of each pixel. In this way, the bound water with its very short relaxation time (about 3 ms) should essentially be visible in the first (odd) and the second (even) echoes, which negligibly (by less than 1%) affects the amplitude deduced from our exponential fit. As a consequence, we can consider that the NMR signal measured corresponds essentially to free water. For this reason, we choose not to consider the first and second echoes in our data process (i.e., numbers of echoes considered from 3 to 16). The validity of this approach was further checked on a system associating two copper sulfate solutions, with respective *T*_2_ values of 4 and 120 ms, similar to bound and free water in wood. This approach makes it possible to obtain, in addition the total water profiles (see above), the 1D profiles of free water in time.

## Appendix

View/request a protocol for this paper from *Bio-protocol*.
